# Mitochondrial and metabolic dysfunction of peripheral immune cells in multiple sclerosis

**DOI:** 10.1186/s12974-024-03016-8

**Published:** 2024-01-20

**Authors:** Peng-Fei Wang, Fei Jiang, Qiu-Ming Zeng, Wei-Fan Yin, Yue-Zi Hu, Qiao Li, Zhao-Lan Hu

**Affiliations:** 1 Department of Anesthesiology, The Second Xiangya Hospital, Central South University, 139 Ren-Min Central Road, Changsha City, 410011 Hunan China; 2 Department of Neurology, Xiangya Hospital, Central South University, Changsha City, 410011 Hunan China; 3 Department of Neurology, The Second Xiangya Hospital, Central South University, 139 Ren-Min Central Road, Changsha City, 410011 Hunan China; 4 Clinical Laboratory, The Second Hospital of Hunan University of Chinese Medicine, 233 Cai’ e North Road, Changsha City, 410005 Hunan China

**Keywords:** Mitochondrion, Immune-metabolic, Immune cells, MS

## Abstract

Multiple sclerosis (MS) is a chronic autoimmune disorder characterized by the infiltration of inflammatory cells and demyelination of nerves. Mitochondrial dysfunction has been implicated in the pathogenesis of MS, as studies have shown abnormalities in mitochondrial activities, metabolism, mitochondrial DNA (mtDNA) levels, and mitochondrial morphology in immune cells of individuals with MS. The presence of mitochondrial dysfunctions in immune cells contributes to immunological dysregulation and neurodegeneration in MS. This review provided a comprehensive overview of mitochondrial dysfunction in immune cells associated with MS, focusing on the potential consequences of mitochondrial metabolic reprogramming on immune function. Current challenges and future directions in the field of immune-metabolic MS and its potential as a therapeutic target were also discussed.

## Introduction

Multiple sclerosis (MS) is a chronic multifocal demyelinating neuroinflammatory disease characterized by progressive neurodegeneration and an autoimmune response [1, 2]. According to the latest Multiple Sclerosis Atlas, 2.8 million people have MS worldwide [3]. MS occurs at a younger age compared to other neurological illnesses [4], with a mean age of diagnosis at 32 years. The prevalence of MS is higher in females, with a female-to-male ratio of 3:1 [5]. MS can be divided into three clinical manifestations: the relapsing–remitting (RRMS), the secondary progressive (SPMS) and the primary progressive (PPMS) [3]. RRMS is the most common form, and it would experience unpredictable onset of neurological symptoms (relapses) and then complete or partially recovery (remissions). Over time, relapsing–remitting attacks become less frequent and neurological function may gradually deteriorate to SPMS. On the other hand, PPMS patients do not experience attacks or remissions, and symptoms continually worsen [3].

Currently, the diagnosis of MS primarily relies on magnetic resonance imaging (MRI), which detects lesions in two or more areas of the central nervous system (CNS) along with corresponding clinical symptoms [6]. Cerebrospinal fluid (CSF) analysis and intrathecal immunoglobulin G production can aid in the diagnosis [7, 8]. However, there has been currently no reliable or sensitive biomarker in peripheral blood to identify the condition. In addition, the majority of existing medications utilized for the treatment of MS primarily improve the patient’s condition and slow the advancement of the disease. Hence, it is imperative to identify early diagnostic markers for diagnosis and efficacious treatments.

Genetic, environmental, and immune factors play significant roles in the development of MS. More than 230 genetic variations associated with a higher risk of developing MS have been identified [9, 10]. Genome-wide association studies (GWAS) provide biological insights into the genetic susceptibility of MS, suggesting that peripheral immune cells are essential mediators of disease risk variants [11]. Some of these variants are related to the aspect of myelin structure or mitochondrial function [12].

A specific genetic factor strongly linked to MS development is a variation in the human leukocyte antigen complex known as HLA-DRB1*15:01. This variation plays a significant role in antigen presentation by antigen-presenting cells (APCs) [10, 13]. Additionally, environmental influences such as obesity, gut microbiota imbalance, virus infection, and smoking contribute to increased risks of developing MS, with Epstein–Barr virus (EBV) infection increasing the risk by 32-fold [14]. These factors may affect mitochondrial biogenesis and functions [15]. Moreover, immune dysregulation in MS leads to the infiltration of immune cells into the CNS, triggering demyelination, axonal damage, and neurodegeneration [16]. The primary target of immune cell attack is the myelin sheath in the white matter of the CNS [17, 18]. Local inflammation and demyelination lead to the diffusion of self-antigens, sequestration of myelin, and activation of autoreactive T lymphocytes [19, 20]. B cells primarily present self-antigens to T lymphocytes through the secretion of self-reactive antibodies [21]. Each immune cell type has a unique metabolic profile crucial for its function and maintenance [22]. Understanding these metabolic profiles may provide new insights into controlling the immune response in MS.

Mitochondria are double-membrane organelle in eukaryotic cells and they are the main site of cellular aerobic respiration. Mitochondria produce adenosine triphosphate (ATP) through oxidative phosphorylation, which occurs in the inner mitochondrial membrane. The electron transport chain (ETC) complex in this process transfers electrons to O_2_ to form reactive oxygen species (ROS). Additionally, other mitochondrial processes such as fatty acid metabolism and tricarboxylic acid cycle (TCA) can also promote ROS production. But if the production of ROS exceeds the physiological limit, it can potentially damage cellular components [23]. Mitochondria also play a role in apoptosis, regulation of calcium balance, production of mitochondrial DNA (mtDNA), oxidative phosphorylation (OXPHOS), and mitochondrial metabolism [24, 25]. Mitochondria significantly contribute to the pathogenesis of MS [26–29] by: (1) accumulating mutations and repairing mtDNA damage; (2) exhibiting abnormal mitochondrial protein or gene expression; (3) displaying defects in mitochondrial enzyme activity and aging; (4) experiencing disturbances in mitochondrial dynamics and changes in the TCA cycle; (5) initiating mitochondria-mediated apoptosis. Elevated glucose and lactate metabolism in active MS lesions have been revealed through investigations using magnetic resonance spectroscopy and positron emission tomography imaging and immunohistochemistry staining [30, 31]. Further evidence of increased metabolic activity in active MS lesions is derived from the higher quantity and functioning of mitochondria in responsive axons [32]. In chronic inactive areas of MS lesions, both the activity of mitochondrial respiratory chain complex IV and mitochondrial mass increase in demyelinated axons [33].

This review summarized the immunological-metabolic mechanisms involved in mitochondrial dysfunction in peripheral immune cells associated with MS. The relationship between MS and mitochondrial function is discussed, with a focus on disease-modifying therapies (DMTs). The aim is to provide insightful hints for investigating the mitochondrial immune function related to MS.

## The role of peripheral immune cells in MS pathology

### Autoimmune response

**Fig. 1:**
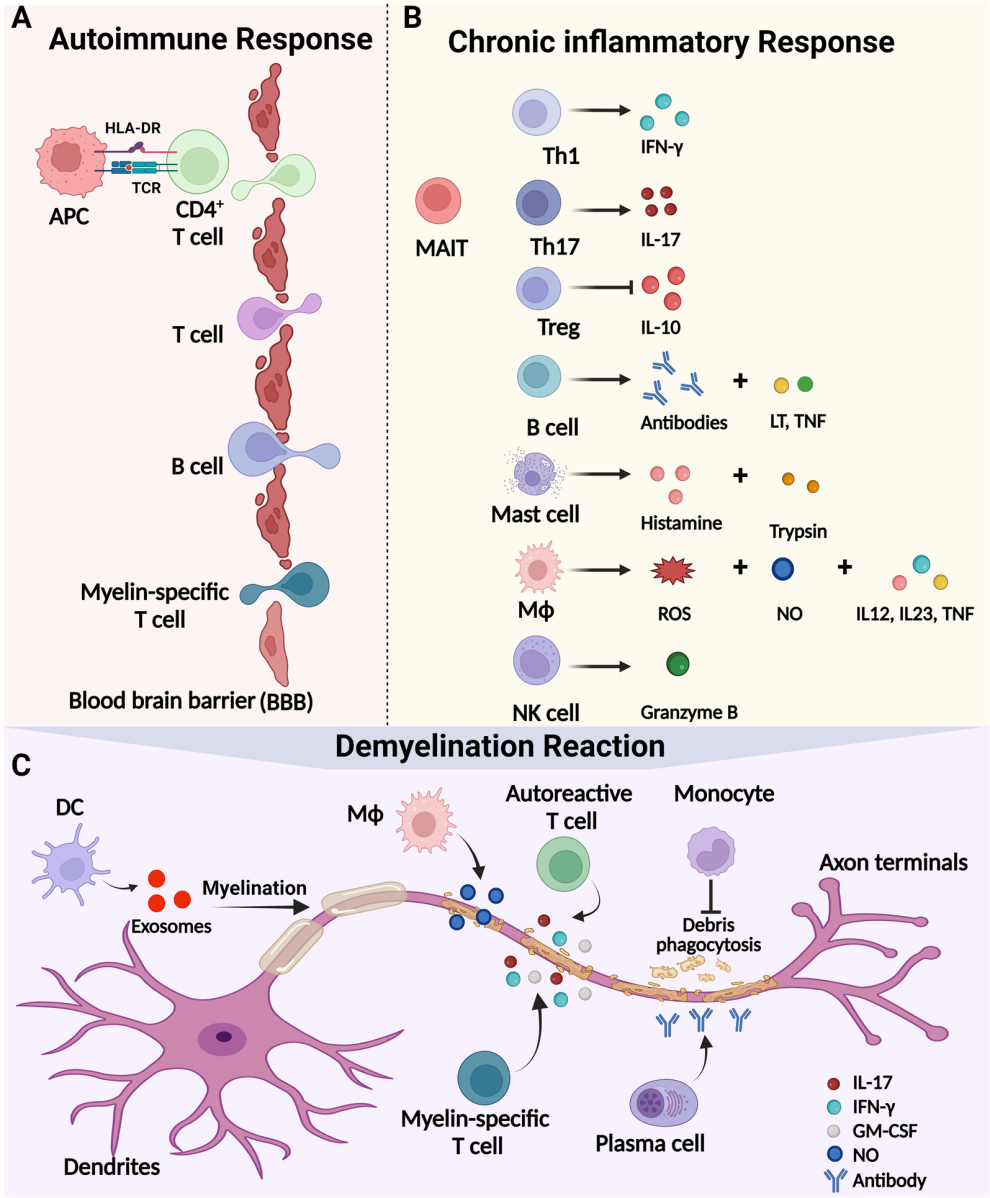
Pathological manifestations of peripheral immune cells in MS. The pathogenesis of MS goes through three main phases: the autoimmune response (**A**), the chronic inflammatory response (**B**), and the demyelinating reaction (**C**). During the autoimmune reaction stage (**A**), various immune cells, such as T cells, B cells, and myelin-specific CD4^+^ T cells, penetrate the brain tissue through the blood–brain barrier (BBB). In the chronic inflammatory response (**B**), adaptive Th cells, Treg, and B cells release cytokines or interferon-γ and antibodies to contribute to the inflammatory response. Additionally, innate immune macrophages (Mϕ), and natural killer (NK) cells secrete substances like histamine, trypsin, ROS, NO, inflammatory cytokines, and Granzyme B, which participate in the inflammatory response. Peripheral immune cells, particularly T cells, B cells, monocytes and Mϕ, contribute to the demyelination process in MS through direct interactions with oligodendrocytes, the release of pro-inflammatory molecules, and the production of antibodies against myelin proteins. MS monocytes inhibit the phagocytic capacity of myeline debris, whereas exosomes derived from DCs promote myelination (**C**)

The autoimmune response is considered a key pathogenic mechanisms underlying MS, wherein an abnormal immune response targets self-antigens within the CNS [34]. This response is triggered by the activation of autoreactive T cells and B cells, which recognize and attack myelin and other components of the CNS [2, 35]. Activated myelin-specific CD4^+^ T cells that react to myelin antigens are primarily responsible for this reaction [36]. Myelin antigens, together with HLA class II molecules and accessory molecules on the surface of APCs, reactivate CD4^+^ T cells in their native environment. The presence of autoantibodies against myelin proteins in the serum and CSF of MS patients indicates that the immune system perceives these proteins as foreign entities and initiates an immune response against them. [37, 38]. Additionally, the presence of inflammatory cells, such as T cells and B cells within MS lesions demonstrates an ongoing immune response within the CNS [39]. Reactivation of MS triggers the release of inflammatory cytokines and soluble mediators, which disrupts the blood–brain barrier (BBB), stimulates chemotaxis, and leads to a larger-scale influx of inflammatory cells into the CNS [40] (Fig. 1A). The experimental autoimmune encephalomyelitis (EAE) model, induced by immunizing animals with myelin-derived proteins (or polypeptides), such as myelin oligodendrocyte glycoprotein (MOG), proteolipid protein (PLP), and myelin basic protein (MBP), serves as a classic animal model of MS. EAE mice model is characterized by the myelin-reactive CD4^+^ T cells and B cells [35, 41].

### Chronic inflammatory response

The chronic inflammatory response in MS is characterized by the activation and proliferation of T cells, B cells, and other immune cells, resulting in the release of pro-inflammatory cytokines, chemokines, ROS and nitric oxide (NO) [16] (Fig. 1B). This chronic inflammation leads to the destruction of myelin, axons, and oligodendrocytes, ultimately causing neurological dysfunction and disability [42]. Recent studies have implicated the innate immune system in the pathogenesis of MS. Activation of toll-like receptors (TLRs) activation leads to the release of pro-inflammatory cytokines and chemokines, contributing to the chronic inflammatory response in MS [43]. Pro-inflammatory Th1 and Th17 have been associated with the pathology of MS [44]. On the contrary, regulatory T cell (Treg) mediates the dysregulation of T cell response in MS by reducing their number and activity [45, 46]. T cells and B cells can be detected in damaged white and gray matter [47]. In MS, B cells differentiate into memory cells or plasma cells, which subsequently secrete autoantibodies involved in antibody-dependent cellular cytotoxicity and complement damage. B cells also have the ability to secrete pro-inflammatory cytokines, including lymphotoxin (LT) and tumor necrosis factor (TNF)-α, significantly contributing to T cell activation [48]. Mucosal-associated invariant T (MAIT) cells, a subset of innate-like CD8^+^ T cells, express semi-invariant T cell receptors as well as accumulate in the MS brain and produce IL-17 [49]. Innate immunity involves mast cells, macrophages (Mϕ) and natural killer (NK) cells. Mast cells in MS plaques release histamine and trypsin simultaneously to facilitate BBB opening [16]. Mϕ produce cytokines such as interleukin (IL)-12, IL-23, and are also involved in the clearance of demyelinating debris [16]. Some subgroups of microglia and Mϕ produce TNF-α, ROS and NO in the activated state, exerting direct neurotoxic effects. CD56^bright^ NK cells promote the production of granzyme B, which is involved in cytotoxicity and autologous CD4^+^ T cells proliferation [50].

### Demyelination reaction

Peripheral immune cells are activated and enter the CNS where they contribute to the destruction of the myelin sheath [51] (Fig. 1C). Myelin-specific T cells exacerbate demyelinating lesions in CNS autoimmunity by releasing cytokines such as IL-17, interferon (IFN)-γ, and granulocyte macrophage-colony stimulating factor (GM-CSF) [52, 53]. Besides, autoreactive T cells mistakenly target and attack myelin, which is the protective covering of nerve fibers in the CNS. These activated T cells release inflammatory cytokines and chemokines that attract other immune cells to the site of inflammation, leading to the further damage to the myelin sheath [54, 55]. In progressive MS, cortical demyelination is associated both with B cell-rich structures in the meninges and plasma cell accumulation in experimental CNS inflammation [56–58], which may be involved in secretory products independent of antibodies, and multiple cytokines produced by B cells in progressive MS patients are cytotoxic to oligodendrocytes and neurons [59, 60]. On the other hand, autoreactive B cells can differentiate into plasma cells, producing antibodies that bind to the myelin sheath and oligodendrocyte proteins. These bound antibodies result in the induction and activation of the complement proteins on tissue surfaces, directly damaging the myelin sheath and exacerbating demyelination [61, 62]. Inflamed monocytes derived from individuals with MS exhibit a diminished ability to engulf and remove myelin debris, a process crucial for prompt and effective remyelination [63]. Mϕ release a substantial quantity of cytokines and NO, penetrating the CNS, which is a vital element in initiating the demyelination response in MS [64]. Distinct subgroups of Mϕ exert contrasting influences on myelination, with inflammatory Mϕ facilitating demyelination [65] and immune-modulatory Mϕ facilitating remyelination [66]. Dendritic cells (DCs) culture stimulated with low-level IFN-γ-released exosomes can increase myelination and reduce oxidative stress both in vitro and in vivo in EAE mice [67].

## The mitochondrial of peripheral immune cells in MS

### Mitochondria in MS T cells

The modulation of T lymphocyte homeostasis in MS is influenced by mitochondrial involvement. Several mitochondrial proteins, including B cell lymphoma 2 (Bcl2), ocular atrophy 1 (OPA1), prohibitin 2 (PHB2), Sirtuin-3 (SIRT3), mitochondrial metalloendopeptidase OMA1, and autophagy related 5 (ATG5), are closely associated with mitochondria-mediated death in MS T cells, probably caused by oxidative stress [29, 68–72]. Some studies have shown that T cells from MS patients exhibit altered mitochondrial structure, reduced glycolysis, downregulated expression and activity of OXPHOS subunits, and decreased mitochondrial membrane potential (MMP) [73–77]. Treatment with IFN beta-1α has the potential to restore the diminished glycolysis and mitochondrial respiration activity observed in T cells derived from patients with RRMS. This restorative effect is linked to elevated levels of aldolase, hexokinase 1 (HK-1), enolase 1 (ENO1), glucose transporter 1 (GLUT1), dihydrolipoamide-S-acetyl transferase (DLAT), and dihydrolipoamide-S-succinyl transferase (DLST) production [77]. Another study found that during relapse, RRMS patients exhibited increased extracellular acidification rate (ECAR) and oxygen consumption rate (OCR), which were not observed during the remission when compared to the healthy controls. Teriflunomide treatment could prevent the proliferation of metabolically active high-affinity T cells involved in the progression of RRMS patients during relapse. This effect is associated with a functional downregulation of OCR and ECAR, along with complex III activity of the ETC in activated T cells. However, it has no impact on mitochondrial content or structure [78].

#### Mitochondria in MS CD4^+^ T cells

A study revealed decreased Fas-mediated apoptosis of CD4^+^ CCR5^+^ T cells in PPMS patients [79]. Moreover, activated CD4^+^ T cells in patients with RRMS exhibited increased expression of the GLUT1 protein at the plasma membrane. This upregulation facilitated the uptake of glucose, leading to significant glucose metabolism and subsequent lactate production. Oxidative stress may be associated with this process, as it lowers the protective enzymes superoxide dismutase (SOD) and glutathione peroxidase (GPX), while raising the expression of the protein Hsp70 [75]. Consistently, high glucose intake exacerbates autoimmunity in EAE and promotes the differentiation of Th17 cells through ROS-driven activation of transforming growth factor (TGF)-β signaling in CD4^+^ T cells. However, there were no changes observed in mitochondrial OCR and ECAR in CD4^+^ T cell metabolism [80]. Tregs with a prominent ROS signature in EAE mice were found. Inhibiting ROS with MitoTEMPO in Tregs of EAE mice attenuated the DNA damage response, prevented Treg cell death, and reduced the differentiation of Th1 and Th17 cells [81]. Other findings also indicate that increased ROS during pathogenic Th1 and Th17 cell development could be a potential metabolic target for MS [82].

There were significant differences in lipid species specific to CD4^+^ T cells between MS patients and control subjects [83]. Short-chain fatty acids (SCFAs) and long-chain fatty acids (LCFAs) were shown to be crucial for the differentiation of CD4^+^ T cells during the progression of EAE mice [84, 85]. Oleic acid, a type of LCFA, restores suppressive deficiencies in tissue-resident Tregs from MS patients by enhancing fatty acid β-oxidation [86]. SCFAs, particularly pentanoate, induce metabolic rewiring by increasing mammalian target of rapamycin (mTOR) activity in CD4^+^ effector T cells. This increase leads to higher acetyl-CoA levels, glucose oxidation, IL-10 secretion, OCR, and suppression of IL-17A production. Ultimately SCFAs affect the balance of Th17/Treg and ameliorate the progress of MS/EAE [87–89]. Saturated fatty acid side chain phospholipids (PLs) possess natural anti-inflammatory properties, reducing EAE symptoms by inhibiting the activation of autoreactive CD4^+^ T cells and promoting apoptosis. This effect is achieved through the suppression of Bcl-2-interacting molecule Bim and Bad phosphorylation [90]. Deletion of acetyl-CoA carboxylase 1 (ACC1) in CD4^+^ T cells prevents de novo fatty acid production, inhibits the glycolytic-lipogenic pathway, and protects mice from EAE by reducing the number of IFN-γ^+^ Th17 cells and increasing the number of FOXP3^+^ Tregs in the spinal cord [91]. Atorvastatin, an inhibitor of HMG CoA, inhibits lipid metabolism and suppresses the T cell activation and differentiation of Th1 and Th2 cells. This immune regulation can be reversed by using HMG CoA reductase L-mevalonate [92]. These findings offer valuable insights into how lipid metabolism regulates the delicate balance between tolerance and adverse immune responses [93].

**Fig. 2:**
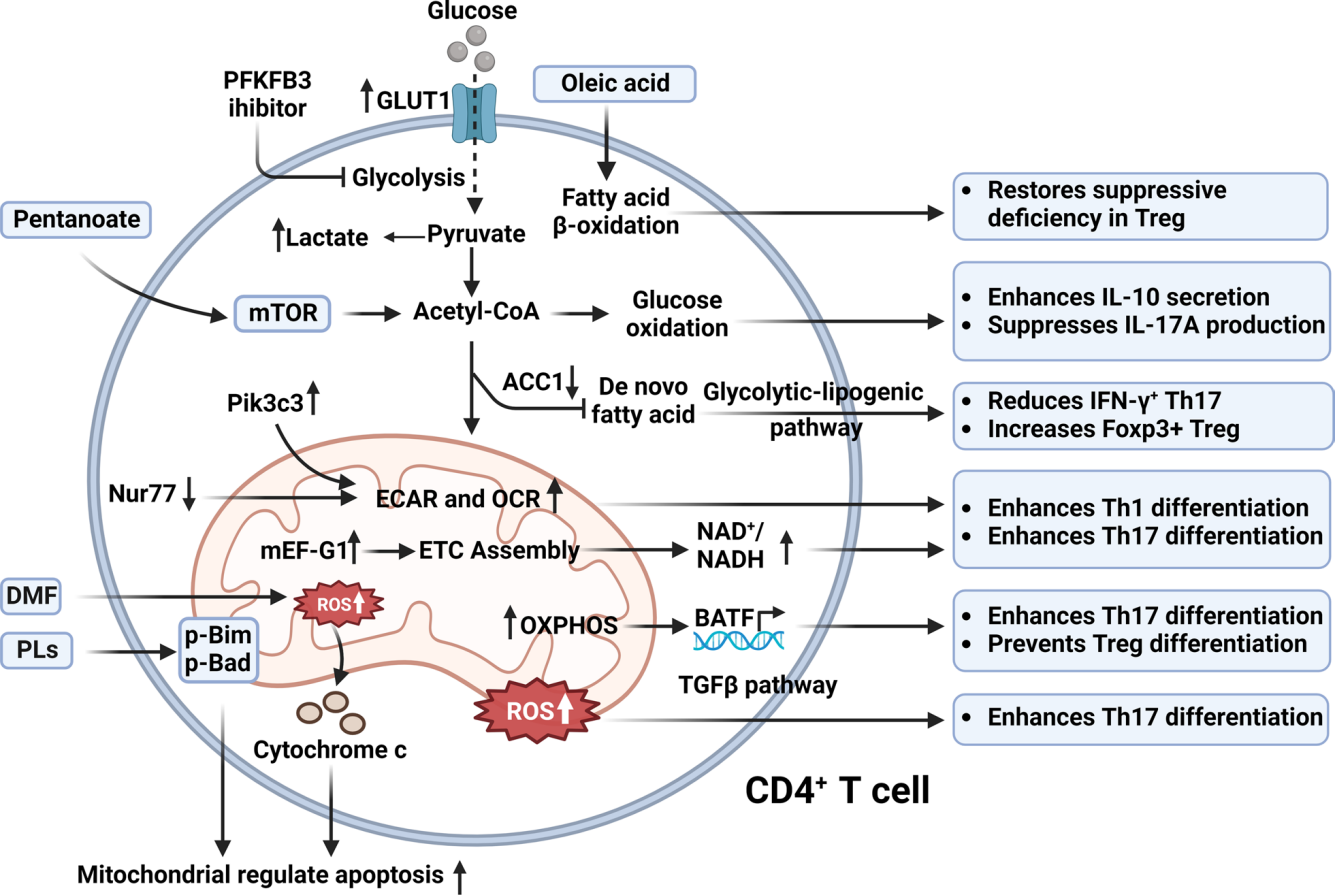
Mitochondria in MS CD4^+^ T cells. CD4^+^ T cells boost the expression of glucose transporter (GLUT)1, resulting in enhanced glucose uptake and lactate generation, which can be blocked by 6-phosphofructo-2-kinase/fructose-2,6-biphosphatase 3 (PFKFB3) inhibitors. The deletion of acetyl-CoA carboxylase 1 (ACC1) reduces the synthesis of de novo fatty acids, decreases IFN-γ^+^ Th17 via the glycolytic-lipogenic pathway, and increases Foxp3^+^ Treg. Pentanoate acts on acetyl-CoA through the mTOR pathway, leading to increased glucose oxidation, secretion of IL-10, and inhibition of IL-17A production. Oleic acid restores the inhibitory state of CD4^+^ to Treg by promoting fatty acid β-oxidation. Pik3c3 increasing and Nur77 deleting both contribute to the elevation of mitochondrial extracellular acidification rate (ECAR) and oxygen consumption rate (OCR). Upregulation of mEF-G1 caused electron transport chain (ETC) assembly in mitochondria, and then elevated NAD^+^/NADH ratio. Above three mechanisms enhance Th1 or Th17 differentiation. Additionally, increased mitochondrial ROS can promote Th17 differentiation. Upregulated mitochondrial oxidative phosphorylation (OXPHOS) influences basic leucine zipper transcription factor TF-like (BATF), promoting Th17 differentiation and inhibiting Treg differentiation. DMF as well as the Bim and Bad pathways of phospholipids (PLs) treatment induces increased apoptosis by augmenting mitochondrial ROS production

Inhibitors of 6-phosphofructo-2-kinase/fructose-2,6-biphosphatase 3 (PFKFB3), which indirectly reduce hypoxia-inducible factor 1α (HIF-1α) activation, have been shown to inhibit Th17 differentiation and exert beneficial effects on MS patients by regulating glycolysis as the main regulator [94]. Upregulated ATP-linked OXPHOS in naïve CD4^+^ T cells can promote Th17 cell differentiation in EAE/MS through decreased expression of aids basic leucine zipper transcription factor TF-like (BATF). Otherwise, this state will switch to Treg differentiation [95]. Consistently, mice lacking BATF exhibited resistance to EAE due to increased chromatin accessibility of Th17 transcription factors [96]. The upregulation of mitochondrial elongation factor G1 (mEF-G1) is implicated in the mitochondrial translation process, leading to enhanced ETC assembly and an increased intracellular ratio of NAD^+^/NADH in CD4^+^ T cells. This upregulation subsequently promotes the development of Th17 and Th1 cells in MS/EAE. The observed phenotypes showed potential for reversal with the administration of the antibiotic linezolid, which perturbing mitochondrial translation in differentiating T cells [97]. Defective cellular metabolism, including downregulation of ECAR and OCR, prevents class III phosphatidylinositol 3-kinase (Pik3c3)-deficient CD4^+^ T cells from differentiating into Th1 cells and makes them resistant to EAE induction [98]. Deletion of Nur77 enhances OCR and ECAR levels in CD4^+^ T cells, resulting in Th1 and Th17 differentiation and exacerbating EAE progression [99]. Suppression of ATPase activity [95] or the use of PFKFB3 inhibitor [94] or 2-deoxy-glucose (2-DG) [87] to control CD4^+^ T cell glycolysis can similarly modify Th17/Treg differentiation. Altogether, CD4^+^ T cells heavily rely on mitochondrial metabolism and dysfunctions for their differentiation in MS disease. The summarized mitochondrial metabolism scheme of MS CD4^+^ T cells is depicted in Fig. 2.

#### Mitochondria in MS CD8^+^ T cells

CD8^+^ T cells represent another important therapeutic target for MS [100]. Cytotoxic CD8^+^ T cells have been detected in MS plaques, CSF, and demyelinated axons [100]. Lactate, a byproduct of glycolysis, accumulates in the CSF of MS patients [101]. Several investigations have demonstrated that shifts in the metabolic activities of CD8^+^ T cells, including glycolysis and OXPHOS, have diverse regulatory effects on the activation, differentiation, and functionality of these immune cells [102]. A higher mitochondrial mass and MMP have been found in the CD8^+^ T cell subset of RRMS patients, accompanied by increased expression of GLUT1. Treatment with 2-DG, a glucose analogue, leads to reduced activation of CD8^+^ T cell subsets in RRMS patients, as proved by decreased expression of the early activation marker CD69, reduced levels of the high-affinity IL-2 receptor CD25, and lower production of TNF α [103].

The study demonstrates that dimethyl fumarate (DMF) significantly alters the metabolic profile of human CD4^+^ and CD8^+^ T lymphocytes and limits caspase-mediated apoptosis to combat oxidative stress by reducing intracellular glutathione (GSH) levels, which are ROS scavengers. As a result, there is an increase in mitochondrial ROS, MMP levels, and an improvement in Cytc, ultimately resulting in a reduction in mitochondrial OCR [104]. Another study has discovered that the inhibition of IL-17-producing CD8^Mϕ^ (Tc17) cells by DMF relies on ROS, which is attributed to heightened PI3K-AKT-T-BET and IL-2-STAT5 signaling pathways. Additionally, DMF-signaling is partially associated with the inhibition of type I or II histone deacetylases (HDACs) and histone acetylation on the IL-17 locus [105]. Enhancing our understanding of the role of mitochondria-targeted therapy in MS may offer a potential immunotherapeutic strategy to restrict T cell-mediated autoimmunity in the future. Table 1 summarizes the mitochondrial and metabolic dysfunction mechanisms that influence T cell function.

**Table 1:** Mechanisms of mitochondrial and metabolic dysfunction that influence T cell function

Mechanism of mitochondrial and metabolic dysfunction	T cell functions	References
Bcl2, OPA1, PHB2, SIRT3, OMA1 and ATG5 regulate mitochondria-mediated death	Regulate apoptosis of T cells	[29, 68–72]
Increase aldolase, HK-1, ENO1, GLUT1, DLAT and DLST, glycolysis and mitochondrial respiration activity via IFN beta treatment	Restore T cell metabolic remodeling	[77]
Increase oxygen consumption rate (OCR) and extracellular acidification rate (ECAR) through enhancing complex III activity of the ETC after teriflunomide treatment	Prevent the proliferation of T cells	[78]
Reduction in the superoxide dismutase (SOD) and glutathione peroxidase (GPX), as well as an increase in the protein Hsp70 caused by oxidative stress, lead to increase GLUT1 and lactate	Regulate CD4^+^ T cell metabolic reprogramming	[75]
ROS-driven activation of TGF-β signaling in CD4^+^ T cells	Encourage the differentiation of Th17 cells	[80]
MitoTEMPO inhibits ROS in Treg	Decline Th1 and Th17 cells differentiation	[81]
Oleic acid enhances fatty acid β-oxidation of Treg	Enhance inhibition of tissue-resident Treg	[86]
Upregulated mTOR activity increases acetyl-CoA levels and glucose oxidation by pentanoate	Increase IL-10 secretion and suppress IL-17A production in CD4^+^ T cells	[87–89]
Phospholipids (PLs) suppress phosphorylation of Bim and Bad molecules	Inhibit the activation and promotion apoptosis of autoreactive CD4^+^ T cells	[90]
Acetyl-CoA carboxylase 1 (ACC1) promotes de novo fatty acid production and the glycolytic-lipogenic pathway	Increase IFN-γ^+^ Th17 cells number and decrease FOXP3^+^ Tregs number in the spinal cord	[91]
Atorvastatin induces p-STAT6, inhibits p-STAT4 expression and cholesterol synthesis	Promote Th2 cells differentiation, inhibit Th1 and Th 17 cells differentiation	[92]
Inhibit PFKFB3-HIF1α activation	Decline Th17 cells differentiation	[94]
Decrease transcription factor TF-like (BATF) expression and upregulate ATP-linked oxidative phosphorylation (OXPHOS)	Promote Th17 cells differentiation and increase the chromatin accessibility	[95, 96]
Linezolid disrupts the integrity of the ETC by inhibiting mitochondrial elongation factor G1 (mEF-G1) and the ratio of NAD^+^/NADH	Promote Th17 and Th1 cells differentiation	[97]
Pik3c3-deficient CD4^+^ T downregulation of ECAR and OCR	Inhibit Th1 cells differentiation	[98]
Nur77 knock-out enhances OCR and EACR	Promote Th1 and Th17 cells differentiation	[99]
2-DG treatment in CD8^+^ T cells reduces CD69 and CD25	Decrease CD8^+^ T cells activation and TNF α production	[103]
DMF reduces intracellular GSH, CytC and induces ROS, mitochondrial membrane potential (MMP), OCR and caspase-mediated apoptosis	Increase apoptosis of CD4^+^ T cells and CD8^+^ T cells	[104]
DMF increases ROS in Tc17 cells through PI3K-AKT-T-BET and IL-2-STAT5 signaling pathways	Decrease IL-17 production in Tc17 cells and inhibit type I or II histone deacetylases (HDACs) histone acetylation on the Il17 locus	[105]

### Mitochondria in MS B cells

B cells can be found in CNS lesions throughout all stages of MS, primarily localized to the perivascular cuffs. B cells serve as effective APCs expressing costimulatory molecules such as CD40, CD80, and CD86 as well as MHC class II [61, 106]. Compared to myeloid APCs, they exhibit significantly higher efficiency, approximately 10,000 times, in capturing soluble and membrane-tethered antigens and presenting them to T cells [107]. In SPMS patients, mitochondrial damage has been observed around CNS periventricular CD20^+^ B cells [108].

In CSF memory B cells, single-cell RNA sequencing studies have shown an increase in cholesterol production [109]. Obeticholic acid (OCA) is a unique medication that regulates various metabolic processes, including cholesterol production, glucose metabolism, inflammation, and apoptosis [110]. It has been proven the effectiveness of OCA in treating EAE, reducing high-density lipoprotein (HDL) levels, cholesterol, and the number of B cells, while OCA alleviates B cell exhaustion by reducing the expression of programmed cell death protein 1 (PD1) and programmed death ligand 1 (PD-L1) [111]. Evidence suggests that increased levels of glycolytic enzymes, glyceraldehyde 3-phosphate dehydrogenase (GAPDH), and triosephosphate isomerase (TPI), in the CSF may contribute to B cell clonal growth in the CNS of MS patients [112].

In vitro studies have demonstrated that inhibition of mitochondrial respiration decreases B cell activation (but not glycolysis). On the other hand, both mitochondrial respiration and glycolysis are involved in B cell proliferation, with a notably higher dependence on mitochondrial respiration. Additionally, the expression of costimulatory molecules CD80 and CD86 is reliant on mitochondrial respiration rather than glycolysis. The dysregulated activation and upregulation of CD80 and CD86 on B lymphocytes from untreated individuals with MS can be effectively counteracted by inhibiting Bruton’s tyrosine kinase (BTKi) as well as modifying metabolic processes. Furthermore, BTKi therapy reduces OCR in circulating B cells, attenuates their activation and antigen presenting potential in vivo, partially through the PI3K/AKT/mTOR pathway [113]. Taken together, these findings indicate that glycolysis, cholesterol metabolism, and mitochondrial respiration play crucial roles in regulating B cell functions associated with the pathophysiology of MS. Table 2 provides a summary of the effects of mitochondrial and metabolic dysfunction pathways on B cell activities.

**Table 2:** The impact of mitochondrial and metabolic dysfunction on B cell functioning

Mechanism of mitochondrial and metabolic dysfunction	B cell functions	References
Downregulated high-density lipoprotein (HDL) production by obeticholic acid (OCA) treatment	Reduce the number of B cells and B cell exhaustion	[111]
Upregulated glyceraldehyde 3-phosphate dehydrogenase (GAPDH) and trisaccharide phosphoisomerase (TPI) in the CSF	Drive B cell clonal expansion	[112]
BTKi treatment limits OCR in B cells partially through PI3K/AKT/mTOR pathway	Attenuate B cell activation and antigen-presenting function	[113]

### Mitochondria in MS monocytes

Monocytes, a type of immune cell, have been shown to influence mitochondrial function in patients with MS. They play a significant role in the demyelination process observed both in MS and EAE and they exhibit high adaptability. Once they infiltrate the CNS during disease progression, monocytes can differentiate into either inflammatory or immune-modulatory macrophages, depending on local conditions. The ratio of these macrophage types ultimately determines the course of MS [114]. Functional enrichment analysis using GWAS datasets revealed a strong association among unique risk genes in monocytes and mitochondria and lipid metabolism [11].

The small molecule inhibitor 6877002 targeting the CD40-TRAF6 pathway alters human inflammatory monocytes, resulting in reduced production of ROS, TNF, and IL-6, while increasing the production of IL-10. This treatment also enhances the ability of monocytes to reduce trans-endothelial migration, which is significant when monocytes infiltrate the CNS during neuroinflammation in EAE [115]. Lactate dehydrogenase (LDH), consisting of lactate dehydrogenase A (LDHA) and LDHB subunits, is a crucial enzyme in the anaerobic glycolysis metabolic pathway implicated in MS pathology [116]. LDHB favors the conversion of lactate to pyruvate, while LDHA, as a rate-limiting enzyme, has a higher affinity for pyruvate, converting it into lactate [117]. Remarkably higher levels of LDHA on CD11b^+^ monocytes in blood from EAE mice have been observed compared to the control condition [118].

Monocytes derived from EAE mice treated with 2-DG display decreased glucose uptake and reduced lactate production in the surrounding medium. Additionally, these monocytes exhibited decreased ECAR, which correlated with a decrease in the expression of key glycolytic enzymes including Glut1, HK-2, TPI, pyruvate kinase M (PKM), LDHA, and monocarboxylate transporter (MCT) 1. Monocytes treated with 2-DG adopted an immune-modulatory phenotype, and once them transplanted into EAE mice, resulted in a significant improvement in disease severity [114]. These findings indicate that glycolysis is highly upregulated in activated monocytes in MS.

**Fig. 3:**
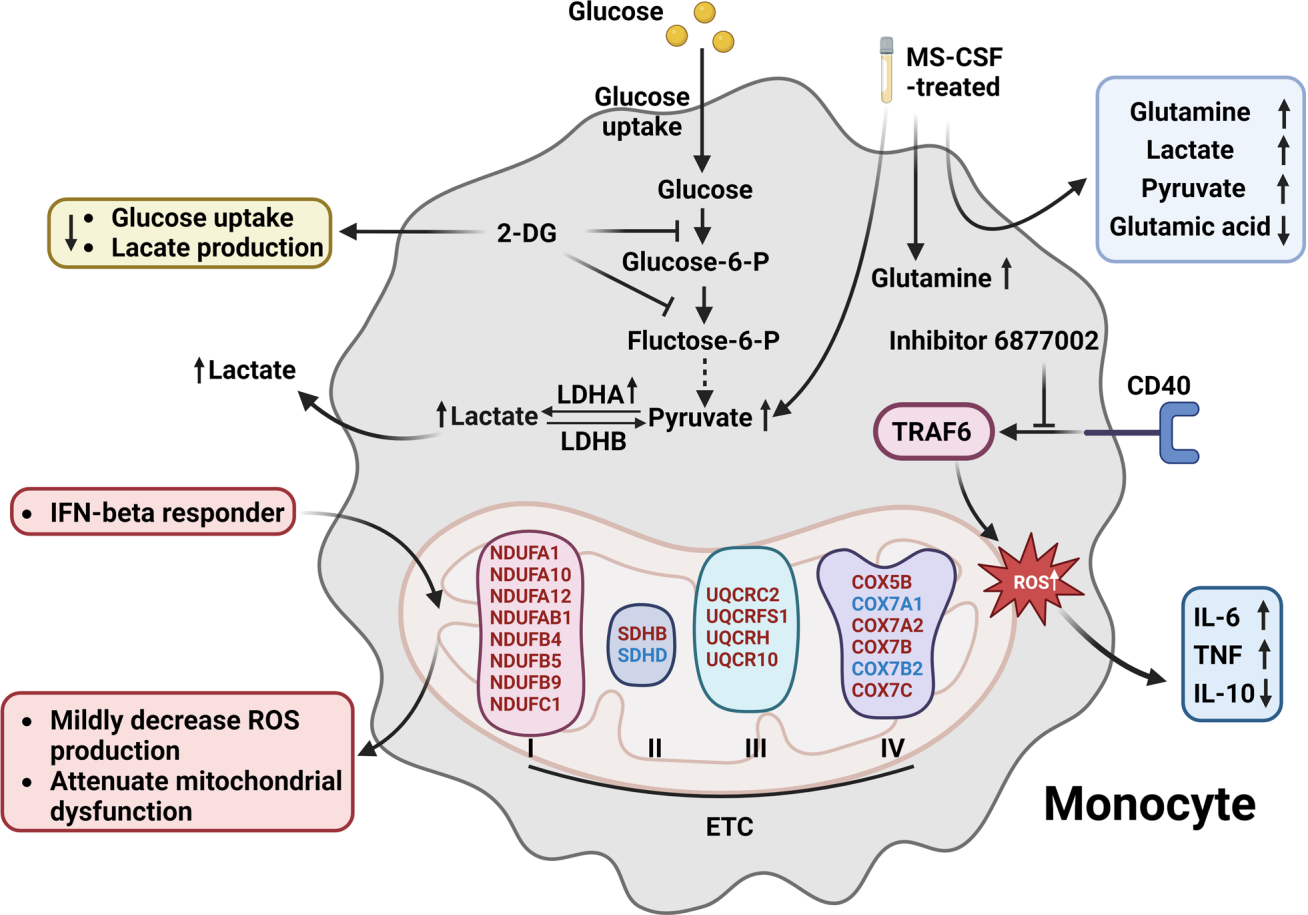
Mitochondria in MS monocytes. Upregulated pyruvate is converted to enormous lactate by increased expression of lactate dehydrogenase A (LDHA) enzyme in MS monocytes. The 2-deoxy-glucose (2-DG) inhibits glycolysis and reduces glucose uptake as well as lactate production. When monocytes are exposed to cerebrospinal fluid (CSF) from MS patients, there is an increase in the production of intracellular glutamine, pyruvate and extracellular glutamine, lactate and pyruvate. While the production of glutamic acid decreases. The small molecule inhibitor 6877002 inhibits the upregulated ROS, extracellular IL-6, tumor necrosis factor (TNF), and decreases IL-10 through the CD40-TRAF6 pathway. MS patients who respond IFN-β treatment alter in electron transport chain (ETC)-related genes, a mildly decrease ROS, and an improvement in mitochondrial dysfunction

Comparison between the MS group and the control group revealed that monocytes treated with MS-CSF produced more pyruvate and glutamine. Extracellular levels of glutamine, lactate, and pyruvate were significantly increased, while levels of glutamic acid were significantly decreased [119]. Quantification of monocyte numbers and assessment of monocytic ROS levels revealed an upregulated trend following DMF administration, allowing for the distinction between responders and non-responders. Mechanistically, a single nucleotide polymorphism (SNP) in *NOX3* (rs6919626 A) is associated with ROS production and responsiveness to DMF treatment [120]. Mitochondrial dysfunction pathways were upregulated in monocytes of individuals who did not respond to IFN-beta treatment. Monocytic ROS production slightly decreased, and several mitochondrial ETC-related genes showed significant alterations in monocytes that responded to IFN-beta treatment compared to non-responders [121, 122]. These results suggest that mitochondrial translation, oxidative stress, and intracellular glycolysis are crucial for monocyte function and pathology, particularly in the context of mitochondrial therapy drugs (Fig. 3). Table 3 provides a summary of the pathways involving mitochondrial and metabolic disorders that impact monocyte activities.

**Table 3:** Mechanisms of mitochondrial and metabolic dysfunction that affect monocyte functions

Mechanism of mitochondrial and metabolic dysfunction	Monocytes functions	References
The inhibitor 6877002 decreases ROS production of monocytes through CD40-TRAF6 pathway	Reduce pro-inflammatory cytokines (TNF, IL-6) production and trans-endothelial migration ability	[115]
2-DG treatment reduces glucose uptake, lactate secretion and ECAR in monocytes, which was correlated with a decrease in the expression of Glut1, HK-2, TPI, PKM, LDHA, and MCT-1	Switch to an anti-inflammatory phenotype and 2-DG treated monocytes attenuate the severity of experimental autoimmune encephalomyelitis (EAE)	[114]
DMF treatment increases ROS production in monocytes, which was correlated with genetic variation and CpG methylation in monocytic *NOX3*	Increase the numbers of monocytes	[120]
IFN-beta treatment alters mitochondrial dysfunction pathway and mitochondrial ETC-related genes	Decrease monocytic ROS production	[121, 122]

### Mitochondria in MS Mϕ

Mϕ actively participate in the immune response to autoimmune diseases and have been implicated in the destruction of myelin and axons in MS lesions [123]. Immunoregulatory Mϕ (M2) drive the remyelination of damaged axons, produce neurotrophic factors, and facilitate oligodendrocyte function [64]. DMF efficiently inhibits antigen-presenting function in Mϕ, suppressing pro-inflammatory mediators such as ROS, inducible NO synthase (iNOS), TNF-α, IL-1β, and IL-6. DMF achieves this by decreasing extracellular signal-regulated kinase (ERK) phosphorylation, promoting M2-like Mϕ in an EAE mouse model [124]. Furthermore, it has been demonstrated that DMF significantly blocks glycolysis by inhibiting GAPDH in murine Mϕ [125]. This may help explain how DMF promotes M2-like Mϕ, considering that M1 Mϕ primarily utilize glycolysis while M2 Mϕ exhibit greater OXPHOS activity [125, 126]. Moreover, FhHDM-1, a 68-mer peptide secreted by the helminth parasite *Fasciola hepatica*, inhibits the progression of EAE/MS by upregulating OXPHOS and decreasing glycolysis in Mϕ. This leads to the inhibition of Mϕ activation and production of pro-inflammatory cytokines, such as TNF and IL-6 [127, 128].

ROS are rapidly produced in the CNS of MS patients, predominantly by activated Mϕ responsible for demyelination and disruption of axons [129, 130]. These oxidative bursts in Mϕ contribute to focal axonal degeneration, which is further exacerbated by mitochondrial damage and iron release from MS lesions [131, 132]. The p47phox subunit of nicotinamide adenine dinucleotide phosphate (NADPH) oxidase, primarily localized within the zone of initial damage or lesions in patients with acute or early RRMS, accumulates in infiltrating Mϕ and mediates ROS generation [133, 134]. RNA sequencing analysis reveals that fibrin induces the core oxidative stress signature (including neutrophil cytosolic factor (Ncf) 2, Sod2, and Nox2) and the p47phox subunit of NADPH oxidase expressed by ROS^+^ Mϕ [135, 136]. In an EAE mouse model, the transcriptomic signature of fibrin indicates a decrease in oxidative stress, demyelination, axonal harm, and prevention of paralysis when mice are exposed to the fibrin-targeting antibody 5B8. This evidence suggests that fibrin plays a significant role in triggering gene programs associated with oxidative stress during the activation of peripheral Mϕ in EAE [135]. Taken together, these findings suggest that the inflammation-associated oxidative burst in Mϕ contributes to demyelination and tissue injury mediated by free radicals in the pathogenesis of MS.

**Fig. 4:**
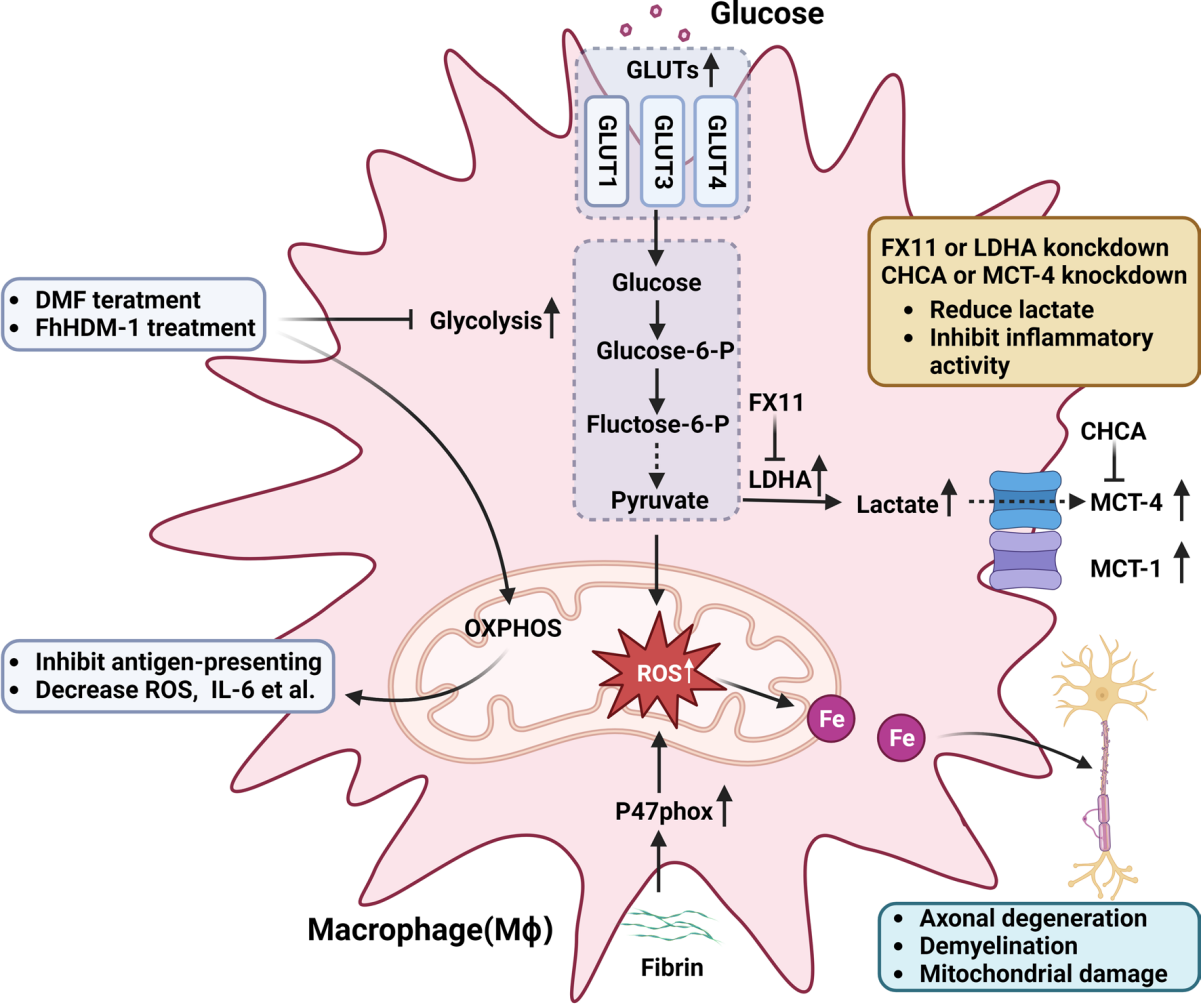
Mitochondria in MS Mϕ. The upregulated glucose transporters (GLUTs) (GLUT1, GLUT3, GLUT4) and monocarboxylate transporter 1 (MCT-1) of Mϕ in patients with MS enhance glycolysis. lactate dehydrogenase A (LDHA)and MCT-4 accumulation in Mϕ also lead to increased lactate production. The 3-dihydroxy-6-methyl-7-(phenylmethyl)-4-propylnaphthalene-1-carboxylic acid (FX11) as well as α-cyano-4-hydroxy-cinnamic acid (CHCA) can reduce lactate production and inflammatory activity. Fibrin induces an increase in p47phox leading to upregulate production of ROS. This is accompanied by elevated release of iron, resulting in axonal degeneration, demyelination and mitochondrial damage. Treatment with DMF or FhHDM-1 can inhibit glycolysis and enhance oxidative phosphorylation (OXPHOS), bringing about decreasing antigen-presenting function and inflammatory mediators such as ROS, IL-6, et al. in MS Mϕ

MCTs play a crucial role in exporting lactate in glycolytic cells [137]. MCT-4 and LDHA are upregulated in inflammatory Mϕ of postcapillary venules in MS patients. Knockdown of LDHA or MCT-4 significantly reduces lactate levels in macrophage supernatant after lipopolysaccharide (LPS) treatment. Additionally, the MCT-4 inhibitor α-cyano-4-hydroxy-cinnamic acid (CHCA) effectively decreases lactate levels and prevents inflammatory activity in Mϕ. The LDHA inhibitor 3-dihydroxy-6-methyl-7-(phenylmethyl)-4-propylnaphthalene-1-carboxylic acid (FX11) also exhibits lactate-reducing properties in Mϕ, inhibiting their inflammatory function [118]. Another study shows that enhanced glycolysis in infiltrating MHC-II^+^ Mϕ leads to increased expression of glucose transporters (GLUTs) such as GLUT1, GLUT3, GLUT4, and MCT1, actively contributing to demyelination in MS lesion tissue [138]. These findings indicate that elevated glycolysis is partially associated with increased expression of glycolytic kinase proteins, which are integral to inflammatory macrophage activation and contribute to demyelination in MS lesions (Fig. 4). The impact of mitochondrial and metabolic dysfunction mechanisms on Mϕ functions is summarized in Table 4.

**Table 4:** Mechanisms of mitochondrial and metabolic dysfunction influence Mϕ functioning

Mechanism of mitochondrial and metabolic dysfunction	Mϕ functions	References
DMF blocks the glycolysis process and ROS production by decreasing extracellular signal-regulated kinase (ERK) phosphorylation	Inhibit antigen-presenting function in Mϕ and switch to M2 phenotype	[124, 125]
FhHDM-1 upregulates oxidative phosphorylation (OXPHOS) and decreases glycolysis	Inhibit Mϕ activation and pro-inflammatory cytokines expression	[127, 128]
P47phox mediated ROS generation	Accumulate in infiltrating Mϕ and involved in demyelination	[133, 134]
Fibrin upregulates ROS production through enhancing oxidative stress genes (Ncf2, Sod2, Nox2) and p47phox	Promote activation of Mϕ and demyelination and axonal damage	[135, 136]
Deleting LDHA or MCT4 reduces lactate production	Inhibit pro-inflammatory Mϕ activity	[118]
Glycolysis upregulated in MHC-II^+^ Mϕ was associated with glucose transporters (GLUTs) and MCT-1	Promote MHC-II^+^ Mϕ activity in demyelination	[138]

### Mitochondria in MS neutrophils

Neutrophils, a crucial population of granulocytes, play a key role in initiating inflammatory responses [139]. Neutrophils in MS display an activated phenotype characterized by increased surface expression of TLR-2 and N-formyl-methionyl-leucyl-phenylalanine receptors (fMLPR). Additionally, their adhesion and migration energies are increased [140]. Once neutrophils enter the target site and become activated, they exhibit various effector functions aiming at neutralizing the entry of pathogens. These effector activities include phagocytosis, degranulation, and the secretion of ROS.

The abundance and function of mitochondria in neutrophils have been discussed for a long time. It is believed that instead of involved in ATP synthesis primarily, neutrophil mitochondria appear to have diverse functions including mtDNA copy number and mitochondrial potential [141]. Moreover, a study found no significant differences in intracellular ROS generation between RRMS patients and healthy controls [142]. However, selective deletion of CXCR2 in neutrophils is crucial for downregulating Ncf1. This downregulation leads to the inhibition of ROS and IL-1β production in neutrophils, resulting in attenuated CNS neuronal damage in EAE disease [143]. In case of lacking Socs3, neutrophils demonstrated heightened activation of granulocyte colony-stimulating factor (G-CSF)/STAT3 signaling, resulting in atypical EAE characterized by intensified neutrophil activation and elevated generation of ROS [144]. The impact of mitochondrial and metabolic dysfunction pathways on neutrophil activities is summarized in Table 5.

**Table 5:** Mechanisms of mitochondrial and metabolic dysfunction influencing neutrophil function

Mechanism of mitochondrial and metabolic dysfunction	Neutrophils functions	References
Knock-out CXCR2 reduces ROS production through inhibiting Ncf1 and IL-1β production	Reduce the inflammatory cytokines	[143]
Deficiency of Socs3 elevates ROS through activating G-CSF/STAT3 signaling	Enhance neutrophil activation	[144]

### Mitochondria in MS DCs

DCs, a type of APCs, are divided into conventional DCs (cDCs) and plasmacytoid DCs (pDCs) [145]. pDCs produce a large amount of type I IFN, which plays a role in antiviral innate immunity and exists in the blood as immature cells [146]. The number of cDCs and pDCs is significantly upregulated in the CSF of MS patients [147, 148]. In EAE, pDCs promote Tregs expansion and inhibit the development of CD4^+^ T cells into Th1 and Th17 cells [149, 150]. Interferon beta-treated DCs induce the gene expression of IL-12p35 and IL-27p28, which suppress the differentiation of Th17 cells and induce IL-10 secretion through the activation of STAT1 and STAT3 in MS [151].

Research highlights the importance of sirtuin 6 (SIRT6) in regulating several processes such as DNA repair, inflammation, immunology, and energy metabolism, including glucose and lipid metabolism [152]. Inhibition of SIRT6 attenuates the onset of EAE by reducing DC migration and activation, potentially mediated via metabolic pathways [153]. NADPH oxidase 2 (NOX2) plays a crucial role in regulating the endocytosis of MOG antigen processing in DCs and supports the presentation of MOG antigen to CD4^+^ T cells through LC3-associated phagocytosis (LAP). Deletion of NOX2 in DCs inhibits the myelin peptide presentation of DCs, preventing the recruitment of CD4^+^ cells to the CNS in EAE mice [154]. Activation of HIF-1α by lactate induces NDUFA4L2 and limits the pro-inflammatory activities and activation of DCs. The inhibition of transcription factor X-box binding protein 1 (XBP1) by NDUFA4L2 in DCs plays a crucial role in controlling pathogenic autoimmune T cells in EAE mice. This regulatory mechanism is achieved through the downregulation of oxidative phosphorylation reprogramming and ROS production [155].

Loss of responsiveness to TGF-β receptor II (TGF-βRII) in monocyte-derived DCs (moDCs) results in increased secretion of IL-12 by moDCs, inducing polarization toward an interferon-gamma-producing Th1 phenotype and exacerbating EAE disease. Mechanistically, upregulated IFN-γ increases Nox-2 and ROS production in moDCs during chronic EAE [156]. DMF induces type II DCs by reducing intracellular GSH through the formation of conjugates, leading to upregulation of heme oxygenase-1 (HO-1) levels [157]. GSH acts as an effective scavenger of ROS, while HO-1 is a sensitive heat shock protein that may result in higher ROS production [158, 159]. Additionally, DMF prevents DCs from expressing pro-inflammatory cytokines and costimulatory molecules [160, 161]. In part, the modulation of the GSH-HO-1 pathway by DMF treatment regulates the oxidative stress during DC maturation and antigen-presenting capacity. These findings suggest that targeting mitochondrial activities of DCs holds promise for the treatment of MS. Table 6 provides a comprehensive overview of the impact of mitochondrial and metabolic dysfunction pathways on DC activities.

**Table 6:** The impact of mitochondrial and metabolic malfunctioning pathways on the activities of DCs

Mechanism of mitochondrial and metabolic dysfunction	DC functions	References
Sirt6 inhibition potentially mediates metabolic pathways	Reduce migration and activation of DCs	[152, 153]
Knock-out NOX2 in DCs can inhibit T cell-mediated autoimmune neuroinflammation through LC3-associated phagocytosis (LAP)	Inhibit myelin peptide presentation of DCs	[154]
Increased HIF-1α-NDUFA4L2 signaling inhibits X-box binding protein 1 (XBP1) and leads to downregulating OCR and ROS	Promote pro-inflammatory activities and activation of DCs	[155]
Tgfbr2 insufficiency in moDCs upregulates ROS production via NOX2	Secret more IL-12 in moDCs which induces Th1 polarization and chronic inflammatory demyelination	[156]
DMF partly though GSH-HO-1 pathway regulating oxidative stress	Decrease maturation and antigen‐presenting capacity of pro-inflammatory DCs	[157, 160, 161]

## Conclusion

Metabolic processes such as glycolysis, the TCA cycle, and lipid metabolism have a strong connection with mitochondrial activities. Furthermore, there may exist a reciprocal link among immune metabolism, immune cell activation and mitochondrial function. Metabolic processes and activities play a significant role not only in producing ATP and biosynthetic precursors but also in influencing immunological responses and the functioning of mitochondria in immune cells. However, it is still unclear whether the improvement in abnormal metabolism is primarily caused by the reprogramming of immune activation or mitochondria.

Research has primarily focused on investigating alterations in the morphology, organization, and functionality of mitochondria in immune cells affected by MS. These investigations have explored various mitochondrial metabolic activities, including mitochondrial respiration, ROS production, MMP, TCA metabolic enzymes, and metabolites. Mitochondrial dysfunction manifests with varying severity across different immune cell populations at different stages of MS progression. Furthermore, the presence of abnormal metabolites influences the functionality and maturation of cellular mitochondria, leading to the persistent release of pro-inflammatory cytokines, neurodegeneration, and demyelination, ultimately affecting the progression of MS. Several DMTs targeting mitochondria and metabolites have been shown to enhance the metabolic activity of immune cells in individuals with MS. This intervention has a significant positive impact on the overall disease status, as summarized in Table 7.

**Table 7:** The present study investigates the impact disease-modifying therapy (DMT) medications on several immune cell populations, with a particular focus on the underlying processes including mitochondrial and metabolic dysfunction

DMT drugs	Immune cell type	Mechanism of mitochondrial and metabolic dysfunction	References
Interferon beta	T cell	Reverse the decreased glycolysis and mitochondrial respiration activity by upregulating the expression of aldolase, HK-1, ENO1, GLUT1, DLAT and DLST	[77]
	DC	Suppress the differentiation of Th17 cells and induces IL-10 secretion via activated STAT1 and STAT3	[151]
	Monocyte	Alter monocytic ROS production and mitochondrial ETC-related genes	[121, 122]
Dimethyl fumarate (DMF)	CD4 or CD8	Increase caspase-mediated apoptosis through CytC, reduce intracellular GSH, increase MMP, ROS levels and mitochondrial OCR	[104]
	Tc17	Upregulate ROS in Tc17 cells through PI3K-AKT-T-BET and IL-2-STAT5 signaling pathways	[105]
	Monocyte	Upregulate monocytic ROS production as a result of genetically sustained low level of the promotor methylation of *NOX3*	[120]
	Mϕ	Inhibit glycolysis process and ROS production through decreasing ERK phosphorylation	[124]
	DC	Regulate oxidative stress potentially through modulation GSH-HO-1 pathway	[157, 160, 161]
Teriflunomide	T cell	Enhance OCR and EACR by boosting complex III activity in the ETC	[78]

However, the complex nature of pathophysiological mechanisms underlying mitochondrial metabolism in MS poses challenges for disease diagnosis and treatment. Unfortunately, current research on precision medicines specifically targeting connections within mitochondrial metabolic pathways is deficient. Further investigation into the mitochondrial and metabolic abnormalities of immune cells, utilizing multi-omics combination techniques and high-dimensional single-cell analysis, may enhance our understanding of the pathophysiology of MS.

## Data Availability

Not applicable.

## Abbreviations

MS: Multiple sclerosis
RRMS: Relapsing–remitting
SPMS: Secondary progressive
PPMS: Primary progressive
MRI: Magnetic resonance imaging
CNS: Central nervous system
CSF: Cerebrospinal fluid
GWAS: Genome-wide association studies
APCs: Antigen-presenting cells
EBV: Epstein–Barr virus
ATP: Adenosine triphosphate
ETC: Electron transport chain
ROS: Reactive oxygen species
TCA: Tricarboxylic acid
mtDNA: Mitochondrial DNA
OXPHOS: Oxidative phosphorylation
DMTs: Disease-modifying therapies
BBB: Blood–brain barrier
EAE: Experimental autoimmune encephalomyelitis
MOG: Myelin oligodendrocyte glycoprotein
PLP: Proteolipid protein
MBP: Myelin basic protein
NO: Nitric oxide
TLRs: Toll-like receptors
Treg: Regulatory T cells
LT: Lymphotoxin
TNF: Tumor necrosis factor
MAIT: Mucosal-associated invariant T
Mϕ: Macrophages
NK: Natural killer
IL: Interleukin
IFN: Interferon
GM-CSF: Granulocyte macrophage-colony stimulating factor
DCs: Dendritic cells
Bcl2: B cell lymphoma 2
OPA1: Ocular atrophy 1
PHB2: Prohibitin 2
SIRT3: Sirtuin-3
ATG5: Autophagy related 5
MMP: Mitochondrial membrane potential
HK-1: Hexokinase 1
ENO1: Enolase 1
GLUT1: Glucose transporter 1
DLAT: Dihydrolipoamide-S-acetyl transferase
DLST: Dihydrolipoamide-S-succinyl transferase
ECAR: Extracellular acidification rate
OCR: Oxygen consumption rate
SOD: Superoxide dismutase
GPX: Glutathione peroxidase
TGF: Transforming growth factor
SCFAs: Short-chain fatty acids
LCFAs: Long-chain fatty acids
mTOR: Mammalian target of rapamycin
PLs: Phospholipids
ACC1: Acetyl-CoA carboxylase 1
PFKFB3: 6-Phosphofructo-2-kinase/fructose-2,6-biphosphatase 3
HIF-1α: Hypoxia-inducible factor 1α
BATF: Basic leucine zipper transcription factor TF-like
mEF-G1: Mitochondrial elongation factor G1
Pik3c3: Class III phosphatidylinositol 3-kinase
2-DG: 2-Deoxy-glucose
DMF: Dimethyl fumarate
GSH: Glutathione
HDACs: Histone deacetylases
OCA: Obeticholic acid
HDL: High-density lipoprotein
PD1: Programmed cell death protein 1
PD-L1: Programmed death ligand 1
GAPDH: Glyceraldehyde 3-phosphate dehydrogenase
TPI: Phosphoisomerase
BTKi: Inhibiting Bruton’s tyrosine kinase
LDH: Lactate dehydrogenase
LDHA: Lactate dehydrogenase A
PKM: Pyruvate kinase M
MCT: Monocarboxylate transporter
SNP: Single nucleotide polymorphism
iNOS: Inducible NO synthase
ERK: Extracellular signal-regulated kinase
NADPH: Nicotinamide adenine dinucleotide phosphate
Ncf: Neutrophil cytosolic factor
LPS: Lipopolysaccharide
CHCA: α-Cyano-4-hydroxy-cinnamic acid
FX11: 3-Dihydroxy-6-methyl-7-(phenylmethyl)-4-propylnaphthalene-1-carboxylic acid
GLUTs: Glucose transporters
fMLPR: N-Formyl-methionyl-leucyl-phenylalanine receptors
G-CSF: Granulocyte colony-stimulating factor
cDCs: Conventional DCs
pDCs: Plasmacytoid DCs
SIRT6: Sirtuin 6
NOX2: NADPH oxidase 2
LAP: LC3-associated phagocytosis
XBP1: X-box binding protein 1
TGF-βRII: TGF-β receptor II
moDCs: Monocyte-derived DCs
HO-1: Heme oxygenase-1
